# Psyllium-Husk-Assisted Synthesis of ZnO Microstructures with Improved Photocatalytic Properties for the Degradation of Methylene Blue (MB)

**DOI:** 10.3390/nano12203568

**Published:** 2022-10-12

**Authors:** Syed Nizam Uddin Shah Bukhari, Aqeel Ahmed Shah, Muhammad Ali Bhatti, Aneela Tahira, Iftikhar Ahmed Channa, Abdul Karim Shah, Ali Dad Chandio, Wael A. Mahdi, Sultan Alshehri, Zaffar Hussain Ibhupoto, Wen Liu

**Affiliations:** 1 State Key Laboratory of Organic-Inorganic Composites, Beijing Key Laboratory of Electrochemical Process and Technology for Materials, School of Material Science, Beijing University of Chemical Technology, Beijing 100029, China; 2 Department of Basic Science and Humanities, Dawood University of Engineering and Technology, Karachi 74800, Pakistan; 3 Wet Chemistry and Thin Film Laboratory, Department of Metallurgical Engineering, NED University of Engineering and Technology, Karachi 75270, Pakistan; 4 Department of Environmental Sciences, University of Sindh Jamshoro, Jamshoro 76080, Pakistan; 5 Dr. M.A Kazi Institute of Chemistry University of Sindh, Jamshoro 76090, Pakistan; 6 Department of Chemical Engineering, Dawood University of Engineering and Technology, Karachi 74800, Pakistan; 7 Department of Pharmaceutics, College of Pharmacy, King Saud University, Riyadh 11451, Saudi Arabia; 8 State Key Laboratory of Chemical Resource Engineering, Beijing Advanced Innovation Center for Soft Matter Science and Engineering, College of Chemistry, Beijing University of Chemical Technology, Beijing 100029, China

**Keywords:** psyllium husk, ZnO, hydrothermal method, methylene blue, photodegradation

## Abstract

Wastewater from the textile industry is chronic and hazardous for the human body due to the presence of a variety of organic dyes; therefore, its complete treatment requires efficient, simple, and low cost technology. For this purpose, we grew ZnO microstructures in the presence of psyllium husk, and the role of psyllium husk was to modify the surface of the ZnO microstructures, create defects in the semiconducting crystal structures, and to alter the morphology of the nanostructured material. The growth process involved a hydrothermal method followed by calcination in air. Additionally, the psyllium husk, after thermal combustion, added a certain value of carbon into the ZnO nanomaterial, consequently enhancing the photocatalytic activity towards the degradation of methylene blue. We also investigated the effect of varying doses of photocatalyst on the photocatalytic properties towards the photodegradation of methylene blue in aqueous solution under the illumination of ultraviolet light. The structure and morphology of the prepared ZnO microstructures were explored by scanning electron microscopy (SEM) and powder X-ray diffraction (XRD) techniques. The degradation of methylene blue was monitored under the irradiation of ultraviolet light and in the dark. Also, the degradation of methylene blue was measured with and without photocatalyst. The photodegradation of methylene blue is highly increased using the ZnO sample prepared with psyllium husk. The photodegradation efficiency is found to be approximately 99.35% for this sample. The outperforming functionality of psyllium-husk-assisted ZnO sample is attributed to large surface area of carbon material from the psyllium husk and the synergetic effect between the incorporated carbon and ZnO itself. Based on the performance of the hybrid material, it is safe to say that psyllium husk has high potential for use where surface roughness, morphology alteration, and defects in the crystal structure are vital for the enhancing the functionality of a nanostructured material. The observed performance of ZnO in the presence of psyllium husk provides evidence for the fabrication of a low cost and efficient photocatalyst for the wastewater treatment problems.

## 1. Introduction

Water is an indispensable source for the existence of life [1]. It is universally significant not only for the living beings, but also for various domestic activities such as cooking [2] and washing [3] and, consequently, it is essential for industrial activities. Besides the agricultural sector, water is consumed in the food [4], beverage [5], tanning [6], paper [7], chemical [8], and textile [9] industries, and so on. The textile industry is huge consumer of water, but these industries produce a huge amount of wastewater [10]. The textile effluent carries a variety of very toxic organic compounds that are not only harmful for aquatic life, but also for the human body. The wastewater from the textile industry is rich with various toxic dyes such as methyl orange, methyl blue, rhodamine, and methyl red, etc., [11]. Amongst various dyes, methylene blue is widely used for different purposes in the industry and found to be a very stable dye [12,13]. Therefore, methylene blue is known as a chronic pollutant for our environment and its treatment from wastewater is highly needed. For this purpose, various potential methods were developed for the degradation and removal of methylene [14]. These methods have some technical issues such as being expensive, complicated, and of limited efficiency. Therefore, certain new technologies or modification in these established methods have to be made in order to overcome these limitations. Amongst various techniques, photo catalytic degradation [15] is widely in practice for the degradation of methylene blue under the irradiation of a specific source of photons and photocatalysts. Hence, a variety of potential photocatalysts were prepared and used for the photocatalytic degradation of organic dyes such as zinc oxide, titania, plasmonic metal particles, and hybrid materials [16]. There are many strategies used for the enhancement of photocatalytic activity of ZnO such as using dopants, composite formation, surface modifications, defects engineering, and the use of green chemistry. The doping seems to be very efficient for the development of active photocatalysts based on ZnO for the degradation of dyes [17]. The challenge of doping into ZnO crystal structures is the possibility of a very minor dopant being added into ZnO, and the stabilization of the dopant into ZnO is another challenge. Various efforts were made to degrade MB by using rare earth metals and non-metallic doped ZnO [18,19,20,21]. Furthermore, the use of non-metal semiconductors deposition into co-adsorbent materials including zeolites [22], silica [23], and activated carbon (AC) [24] shows an enhancement of the photocatalytic properties of adsorbent materials. Among them, AC is associated with attractive features such as high surface area, and, consequently, more adsorption capacity into AC is unavoidable [25]. The strategy of using the surface-modifying agents, and likewise an incorporation of AC, is an interesting methodology to use a ZnO-based photocatalyst for the complete removal of organic dyes from the wastewater, although such an approach has not been reported yet. Psyllium husk is used for biological and food ingredient applications [26,27,28]. Psyllium husk is low cost, and its gel-like features in water could lead to altering the surface properties of metal oxides; therefore, enhanced photocatalytic properties are expected. We propose the use of psyllium husk as a potential surface-modifying agent and, at the same time, it adds AC into ZnO during the calcination process, and provides a hybrid photocatalyst for the efficient photodegradation of methylene blue. There is no report on the use of psyllium husk with ZnO for the development of a hybrid material with enhanced photocatalytic properties. Psyllium husk is associated with a wide range of attractive chemical species such as xylose, uronic acid, gardoside, and loganic acid [29,30].

In this study, we applied psyllium husk (Ispaghol) as a surface-modifying agent for tuning the surface and photocatalytic properties of ZnO using a hydrothermal method followed by calcination treatment at high temperature for the incorporation of AC into ZnO, and, consequently, a hybrid photocatalyst was developed. A degradation efficiency of 99.35% was estimated for the removal of methylene blue. The initial dye concentration and catalyst dose were the parameters during the investigation of photocatalytic activity of hybrid materials.

## 2. Material and Methods

### 2.1. Materials

Zinc acetate dihydrate (ZnC₄H₆O), 25% ammonium hydroxide (NH₄OH), methylene blue dye, and ethanol (C_2_H_5_OH) were purchased from Sigma Aldrich Karachi, Sindh, Pakistan. The psyllium husk (Ispaghol) was received from a local medical store located in Jamshoro, Sindh, Pakistan. All the reagents were of high analytical grading and used as received. In typical synthesis, zinc acetate dihydrate (2.224 g) was dissolved in 100 mL of deionized water and also 5mL ammonium solution (25%) was added as a source of hydroxide ions. This mixture was presented in a beaker and designated as pristine ZnO sample. In another mixture, keeping similar amounts of zinc acetate dihydrate and ammonium hydroxide, a surface-modifying agent and carbon source of psyllium husk with a quantity of 1 g in 5 mL of ethanol was added into the precursor solution. The second mixture was labeled as the ZnO/psyllium husk hybrid system. Then, both mixtures were covered with aluminum sheet and placed in pre-heated electric oven (DZF-6050-HT, MTI corporation) at 95 °C for 5 h. At the end, the electric oven was switched off and naturally cooled at room temperature. A white precipitate product in the case of pure ZnO was obtained and filtered out on the filter paper. However, in case of the psyllium husk, three layers were found in the beaker: first was jelly layer, second was a aqueous layer consisting of impurities and solvent, and in the third, the brown product of precipitates was observed. After discarding the first two layers, the brown precipitates were collected on the filter paper and washed several times with ethanol and distilled water. Then, the product was dried at 60 °C for 12 h. Later on, calcination was performed on both pure ZnO and ZnO/psyllium husk hybrid at 250 °C for 2 h. The brown product was transformed into light–dark color containing white material embedded into to it, and was labelled as ZnO/ psyllium husk hybrid material.

### 2.2. Structural and Functional Characterization of as Prepared ZnO Microstructures

Powder X-ray diffraction (Miniflex 600, Rigaku, Austin, TX, USA) was used to identify the phase and pure conditions of different ZnO materials and the set experimental conditions were 45 kV, 45 mA, and CuKα radiation (λ = 1.5418 Å). Scanning electron microscopy with a ZEISS Gemini SEM 500, Gaithersburg, MD, USA, equipped with field emission gun was used to observe the morphology of prepared microstructures, and the measurement was performed at 10 kV as an accelerating voltage. The optical studies of band gap and absorption spectra of dye degradation were performed on a UV–visible spectrophotometer (PE Lamda365, Waltham, MA, USA). The degradation of methylene blue was carried out under the illumination of UV light for a time interval of 20 min. Different photocatalyst doses of ZnO/psyllium husk such as 5, 10, 15, and 20 mg were used within the 10 mg of methylene blue in 500 mL of distilled water. A homemade photo reactor was built and 10 W UV lamps with a wavelength of 365 nm for irradiation of dye solution in the absence and presence of photocatalyst were used. Prior to irradiation light, mechanical stirring was performed on the dye solution containing the photocatalyst in order to maximize the adsorption and desorption processes. The degradation efficiency of the presented photocatalytic process was estimated through the following equation:

(1) $$D\%=\frac{Co-Ct}{Co}\times 100$$

*Co* and *Ct* show the initial dye concentration and dye concentration after certain time, respectively.

The kinetic parameters were obtained using following mathematical representation:

$$\ln\;\left(\frac{ct}{co}\right)=-Kapp\times t$$

*Kapp* shows the rate constant information of degradation kinetics and dye concentration is expressed as mg/L.

To calculate the average crystalline size of pure and ZnO/psyllium husk hybrid samples, the Scherrer equation was followed, as shown:

(2) $$D=\frac{k\lambda }{\beta cos\theta }$$

Here, D represents the average size of crystal, *λ* is the wavelength of X-ray, *k* indicates the value of dimensionless constant for shape factor, and *β* indicates (FWHM intensity), where θ is the Bragg’s angle.

To estimate the band gap of pure ZnO and ZnO/psyllium husk hybrid samples, Tauc’s plot is performed. The absorption coefficient α for direct band gap materials relates to optical band gap energy according to the expression [31].

*αhʋ* = (*hʋ − E_g_*)^1/2^ (3)

Here, *h* is Planck’s constant and *ν* is the frequency of incident photon. The intercept on the photon energy axis is obtained by extrapolating the linear portion of the (*αhν*)^2^ versus *hν* curve.

## 3. Results and Discussion

### 3.1. Composition, Structure, and Morphology Investigations of Psyllium-Husk-Assisted ZnO Microstructures

**Figure 1:**
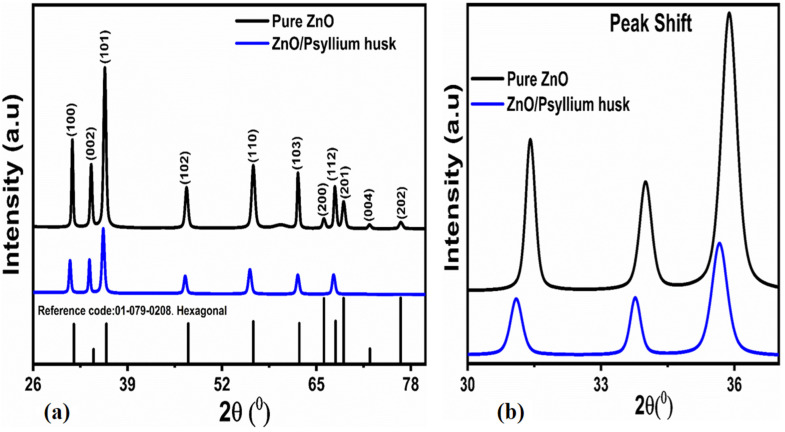
(**a**) XRD diffraction patterns of pure zinc oxide and psyllium-husk-assisted ZnO. (**b**) XRD pattern showing peak shift.

The phase structures of various ZnO microstructures obtained with and without psyllium husk were elucidated by the XRD technique. The diffraction patterns of pure ZnO correspond to (100), (002), (101), (102), (110), (103), (200), (112), (201), (004), and (202) crystal planes and they are fully matched with standard JCPDS card no. 01-079-0208 [32], and a typical wurtzite hexagonal phase is identified. After the use of psyllium husk, ZnO maintains the crystallographic phase of the wurtzite structure and a slight shift in two theta angle such as 31.5°, 34.2°, and 35.8° along the (100), (002), and (101) planes, respectively, is noticed, as shown in Figure 1b. The psyllium-husk-assisted ZnO microstructures show similar diffraction patterns to that of pure ZnO, and correspond to standard JCPDS card no. 01-079-0208. The Scherrer equation was used to estimate the average crystallite size of pure ZnO and psyllium-husk-assisted ZnO, which are enclosed in Table 1. The calculated values of the average crystallite size of ZnO in the case of the pure sample and psyllium-husk-assisted ZnO show that the crystallite size is negligibly affected by the use of psyllium husk during the growth of ZnO. We obtained polycrystalline ZnO for both samples including pure ZnO and psyllium–ZnO microstructures.

**Scheme 1:**
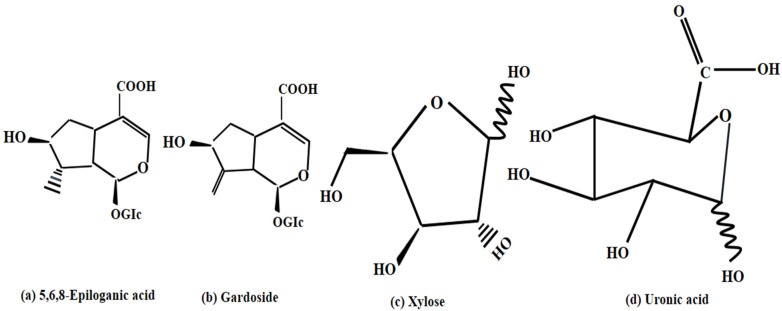
Shows main chemical constituents of psyllium husk.

**Figure 2:**
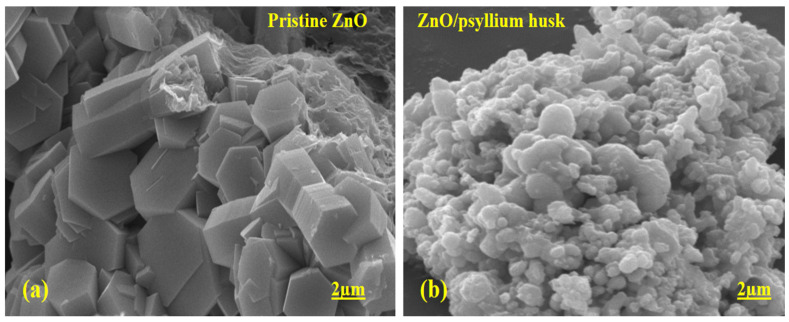
SEM images (**a**) pure ZnO and (**b**) psyllium–ZnO microstructures.

The morphology of the pure ZnO and psyllium-husk-assisted ZnO microstructures was investigated by low-resolution scanning electron microscopy, and the typical SEM images are enclosed in Figure 2. It is obvious from SEM that the distinctive hexagonal facets of nanorod-like morphology are shown by pure ZnO, as depicted in Figure 2a. The microstructure dimensions of microrods for the pure sample of ZnO are in the range of an average diameter of 200–300 nm and the length of few microns. The use of psyllium husk transforms the morphology from hexagonal facets to rough-surface-oriented particle structure, as shown in Figure 2b. From SEM analysis, it is apparent that the psyllium husk has a high potential to alter the surface features of materials; thereby, it could pave the way towards excellent functional properties. It is interesting that the nanostructured dimension is reduced with the use of psyllium husk, and ZnO exhibits the size of 100–200 nm. The psyllium husk used during the growth process is actively involved in the alteration and modification of the surface properties of ZnO, due to presence of a wide range of useful chemical components in the psyllium husk, which act as surface-reducing and modifying agents, as shown in Scheme 1.

**Figure 3:**
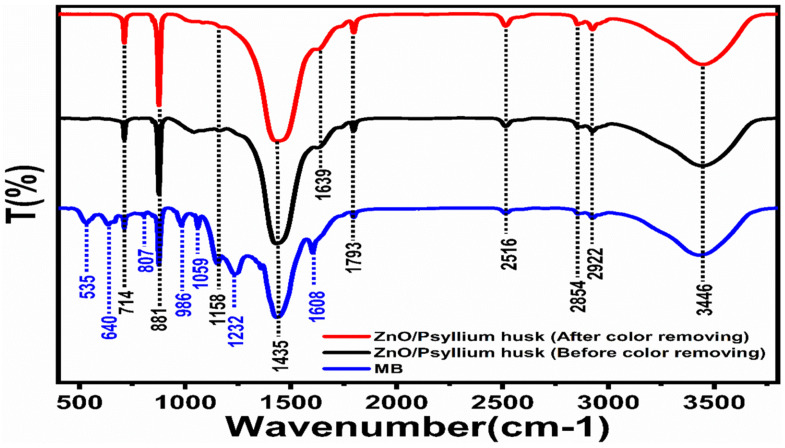
FTIR spectra showing curves for bare MB and ZnO–psyllium husk before and after color removal.

FTIR analysis was performed by a Fourier transform infrared (FTIR) spectrophotometer (Shimadzu, 8400S) for the bare methylene blue, before and after the removal of methylene blue on the surface of psyllium-husk-assisted ZnO microstructures, as shown in Figure 3. In the case of the bare samples of psyllium-husk-assisted ZnO nanostructures, the typical band absorption of metal–oxygen are noticed between 450–600 cm^−1^. For the bare methylene blue, the various characteristic band absorption modes and functional groups are observed. However, after the removal of dye from the surface of psyllium-husk-assisted ZnO nanostructures, the ZnO maintains the typical metal oxide bands, suggesting the stability of material, and many of the vibration bands of methylene blue disappear, thus, revealing the enhanced activity of material for the degradation of dye under the illumination of UV light.

### 3.2. The Photodegradation of Methylene Blue on the Surface of Psyllium-Husk–ZnO Microstructures

**Figure 4:**
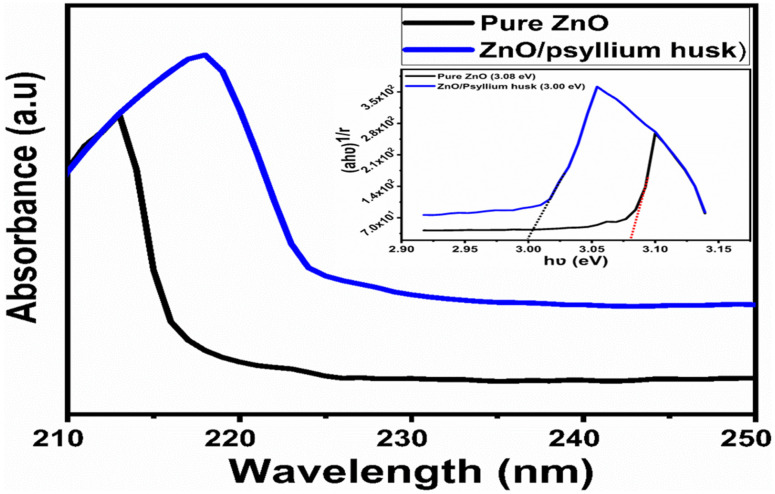
Absorption spectra inset with Tauc plots for pure ZnO and its hybrid with psyllium husk.

The photocatalytic studies were followed by the evolution of the optical band gap for the pure ZnO and ZnO hybrid material produced with psyllium husk, as shown in Figure 4. The Tauc plots indicate the optical band gap for pure ZnO is estimated at 3.08 eV, and for the hybrid material at 3.00 eV. It is reported that the optical band gap for pure ZnO is 3.37 eV [33]. The decrease in the optical band gap could avoid the charge recombination rate, thus, it fosters the degradation efficiency of methylene blue under the irradiation of UV light.

**Figure 5:**
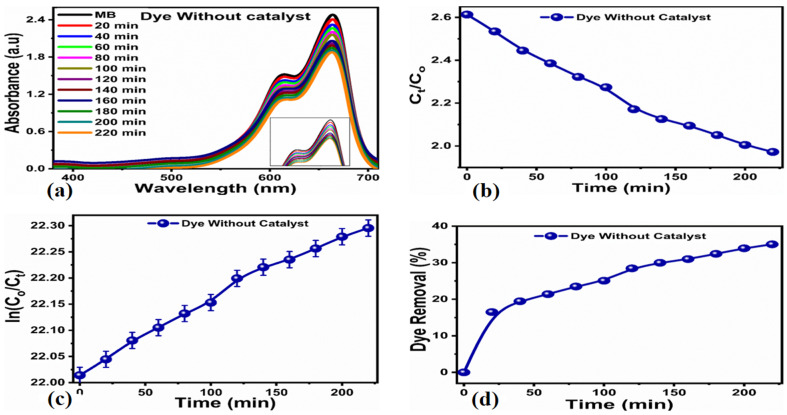
Graphs for MB dye without any catalyst. (**a**) Absorption spectra, (**b**) Ct/Co curve, (**c**) kinetics of degradation, and (**d**) efficiency of dye removal.

**Figure 6:**
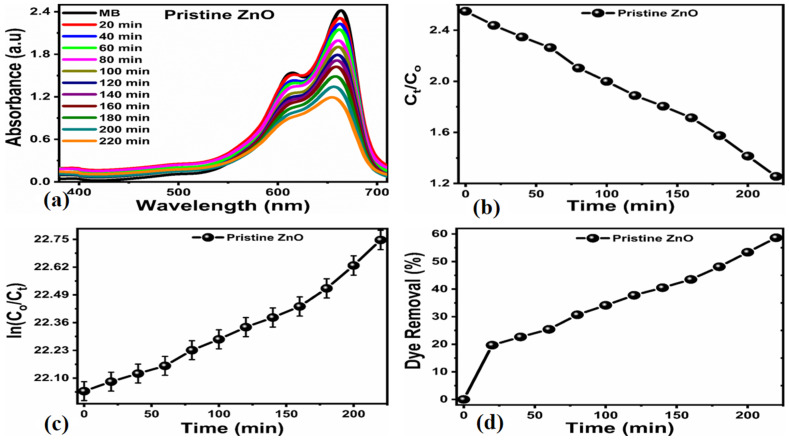
(**a**) Absorption spectra for pristine ZnO, (**b**) Ct/Co vs time curve for, (**c**) kinetics of photodegradation for ZnO towards MB, and (**d**) dye degradation efficiency graph.

Prior to evaluation of psyllium-husk-assisted ZnO microstructures, firstly, the degradation of a bare 2.5 × 10^−5^ M solution of methylene blue dye was examined under the irradiation of UV light. Each degradation measurement was observed with the time interval of 20 min for the total time period of 220 min. A poor degradation efficiency of 35% is noticed for bare dye under the illumination of UV light, as depicted in Figure 5. In the second experiment, the pure ZnO was used as a photocatalyst for the degradation of methylene blue under the illumination of UV light. Similar time intervals were used and a negligible effect on the degradation efficiency is found, as shown in Figure 6.

**Figure 7:**
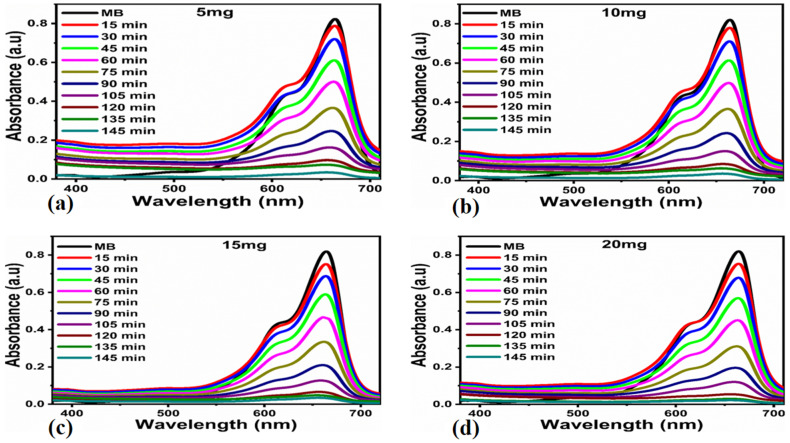
Low concentration absorption spectra under UV irradiation for psyllium-added ZnO with various concentrations of 5 mg (**a**), 10 mg (**b**), 15 mg (**c**), and 20 mg (**d**).

**Figure 8:**
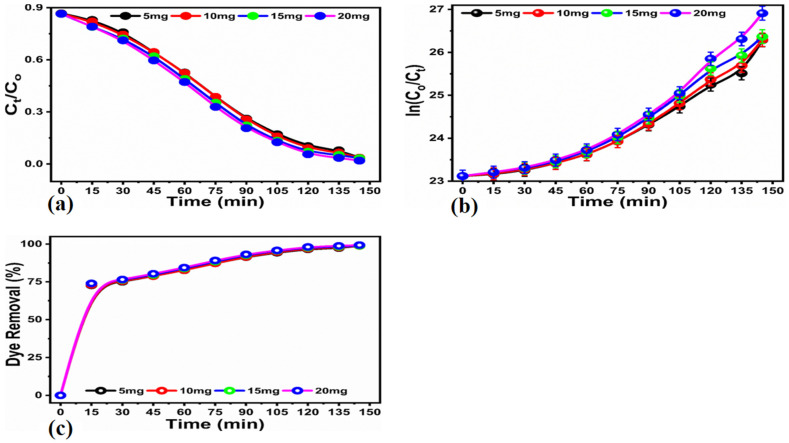
Graphs for various concentrations of 5, 10, 15, and 20 mg of ZnO that contains psyllium: (**a**) Ct/Co curve, (**b**) kinetics of degradation, and (**c**) efficiency of degradation at low concentration.

**Figure 9:**
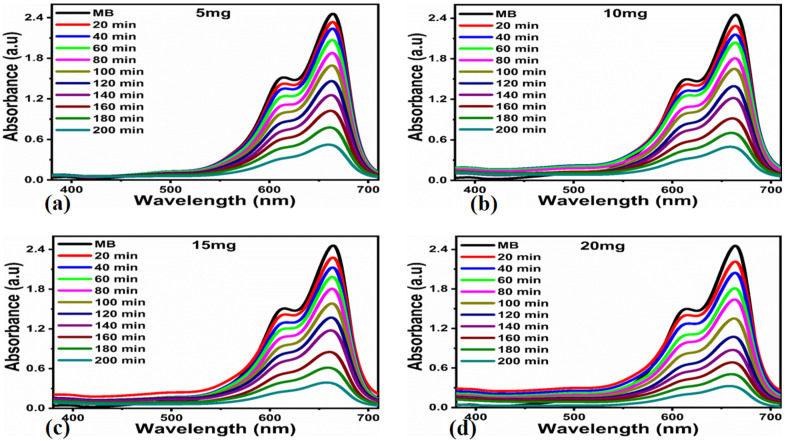
High concentration absorption spectra under UV irradiation for psyllium-added ZnO with various concentrations of 5 mg (**a**), 10 mg (**b**), 15 mg (**c**), and 20 mg (**d**).

**Figure 10:**
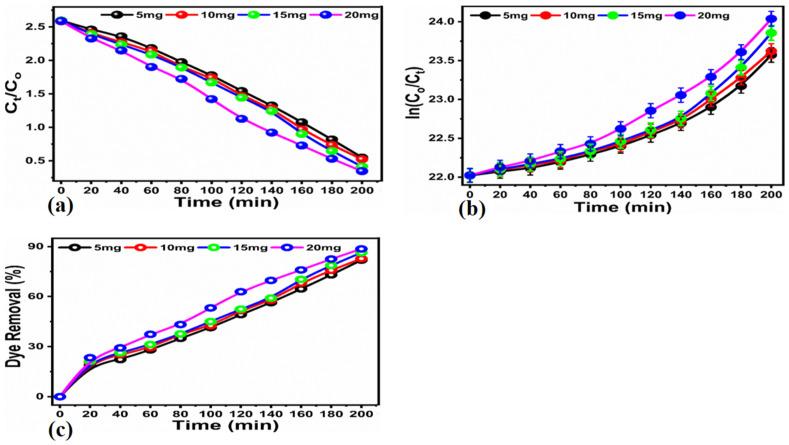
Degradation kinetics for psyllium–ZnO towards removal of MB: (**a**) Ct/Co vs. time, (**b**) ln (Co/Ct) vs. time, and (**c**) dye removal % age vs. time at high concentration.

However, the degradation of methylene blue performed with the ZnO/psyllium husk hybrid shows enhanced degradation kinetics, suggesting a driving role of carbon content due to synergetic effect between carbon and ZnO. The psyllium husk adds certain surface-modifying value in tuning the catalytic properties of ZnO, and the presence of carbon also provides the large surface area for the particles of ZnO. Therefore, the dual role of surface-modifying and carbon incorporation from psyllium husk and catalytic sites from the ZnO together build a synergetic effect that leads to an excellent contribution towards the photodegradation of methylene blue. The degradation of methylene blue was evaluated with different catalyst doses of ZnO/psyllium husk and concentrations, such as 2.5 × 10^−5^ M and 6.5 × 10^−5^ M of methylene blue. The first evaluation of methylene blue degradation was carried out at 2.5 × 10^−5^ M using different quantities such as 5, 10, 15, and 20 mg of ZnO/psyllium husk hybrid under the illumination of UV light for the time period of 150 min, as shown in Figure 7. It is noticed that the psyllium husk plays a significant role in enhancing the photocatalytic properties of ZnO, and the observed degradation efficiencies for the 5, 10, 15, and 20 mg of hybrid catalysts are 98.79%, 98.80%, 98.89%, and 99.35%, respectively, as depicted in Figure 6. The degradation kinetics of methylene blue are depicted in Figure 8a,b, and a visible first order reaction mechanism is demonstrated through the linear plotting (Hosseini et al., 2018). The measured rate constants for various catalyst doses of the first order are enclosed in Table 2. The catalyst dose magically controls the activity of dye degradation, as confirmed by the large value of rate constant of the high catalyst dose. Importantly, the ZnO/psyllium husk hybrid reveals superior photocatalytic activity compare to pure ZnO, and the highest activity is achieved for the optimized value of 20 mg as catalyst dose, as shown in Figure 8c. The increase in performance of the ZnO/psyllium husk hybrid may be attributed to surface roughness, defects in the hybrid material as indicated by XRD, large surface area, and high density of catalytic sites. Similarly, the performance evaluation of methylene blue degradation at higher concentration of 6.5 × 10^−5^ M was performed using various catalyst doses such as 5, 10, 15, and 20 mg with the illumination of UV light for the time period of 200 min, as shown in Figure 9. The UV–visible absorption spectra are depicted in Figure 9. The absorption is sharply decreased even at a higher concentration of methylene blue, indicating the superior photocatalytic activity of the ZnO/psyllium husk hybrid. The first order kinetics as described at a higher concentration of methylene blue are depicted in Figure 10a,b. The calculated rate constant values for the different catalyst doses are enclosed in Table 2, and they indicate the good activity of material even at a high concentration of dye. The relative degradation efficiency of each catalyst dose is 81.87%, 82.75%, 86.34%, and 88.61% for 5, 10, 15, and 20 mg, respectively, as shown in Figure 10c. Based on the performance of the ZnO/psyllium husk hybrid for the catalyst dose 20 mg, it is clear that at a low concentration of dye, the time of degradation is short and the optimal degradation efficiency of 99.35% is achieved. However, the performance of the ZnO/psyllium husk hybrid photocatalyst is even better at higher concentrations of dye, but relatively more time is required in comparison to a low concentration of methylene blue.

### 3.3. Effect of Scavengers

**Figure 11:**
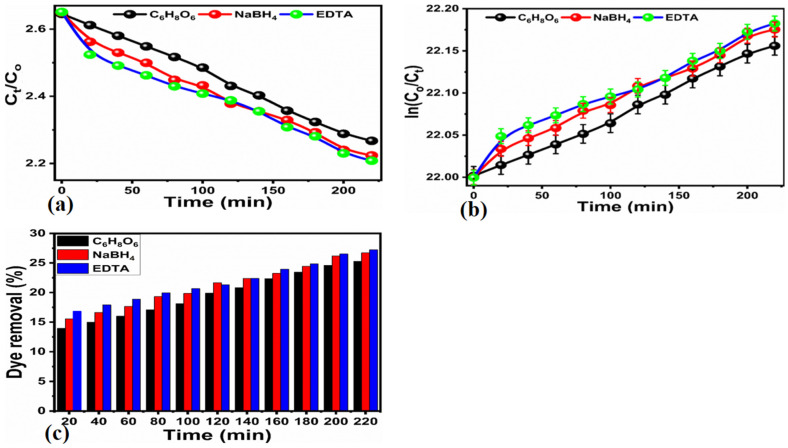
Scavenger study of ascorbic acid (C6H6O8), sodium borohydride (NaBH4), and ethylenediamine tetraacetate (EDTA) for the illustration of reactive species involved in the photodegradation of MB. (**a**) Ct/Co mechanism for scavengers; (**b**) Reaction kinetics for scavengers; (**c**) Removal of dye with respect to time.

For understanding the degradation mechanism, we performed a scavenger study using ascorbic acid (C_6_H_6_O_8_), sodium borohydride (NaBH_4_), and ethylenediamine tetraacetate (EDTA), as shown in Figure 11. They significantly affect the concentration of (O^−2^), and hydroxyl radicals (^•^OH) [34]. It is shown that the degradation of MB is effectively controlled by the oxidizing species [33,34]. This is the reason why we investigated the (C_6_H_6_O_8_), (NaBH_4_), and (EDTA) scavengers for the identification of main oxidizing radicals, and we observe that ascorbic acid has a pronounced effect on the decrease in degradation efficiency; therefore, the hydroxyl radicals are the main radicals involved in the reaction process [35,36]. Furthermore, a kinetics study was also carried out, as shown in Figure 11a,b, and it follows the pseudo first order reaction kinetics, with relatively low values of rate constants compared to the degradation kinetics in the absence of scavengers. The degradation efficiency is also prominently decreased with ascorbic acid compared to other scavenger substances, as shown in Figure 11c.

### 3.4. Photocatalytic Degradation Mechanism

**Scheme 2:**
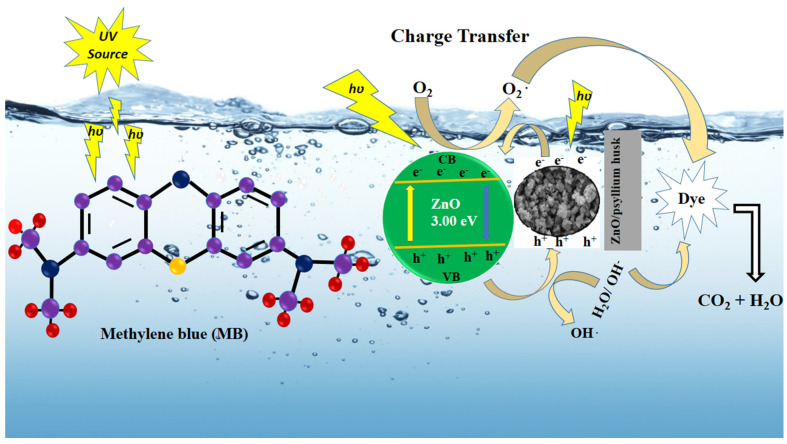
Photodegradation mechanism on ZnO hybrid material produced from psyllium husk.

When the ZnO/psyllium husk hybrid is present within the solution of methylene blue, when treated with a beam of photons with relatively higher energy than the optical band gap of ZnO that enables the excitation of ZnO, then electrons from the valence band of ZnO travel to the conduction band of ZnO, leaving the valence band with a hole. The hole is well-characterized by an active oxidizing agent that can be utilized by the water molecules, and turns water into hydroxyl ions followed by hydroxyl free radicals [37,38]. The presence of exited electrons on the surface of the ZnO hybrid transform the oxygen molecules to superoxide free radicals [39]. In a combined state, the light electrons and holes, and hydroxyl and superoxide free radicals ultimately react with the targeted organic dye through the redox process, then the targeted dye is completely degraded into minerals such as water and carbon dioxide. This is shown in Scheme 2. Another aspect of electron and hole pairs is the recombination with each other or with other photogenerated electrons, or holes with the photocatalysts or at the surface, which severely limits the photodegradation kinetics of the developed photocatalyst [40]. In our case, the reaction mechanism of methylene blue on the surface of the hybrid material is favored by the combined effect of surface electrons, holes, reduced band gap, defects in the structure, alteration in the morphology, high concentration of hydroxyl radicals, and superoxide free radicals. The present work is compared with similar work with respect to other dopants in Table 3.

## 4. Conclusions

In this study, we used psyllium husk as surface-modifying agent for ZnO microstructures during a hydrothermal process and simultaneously added the carbon content followed by a combustion process in air. The surface modification and development of synergetic effect between carbon and ZnO enables the material to demonstrate enhanced photocatalytic properties. The psyllium husk transforms the ZnO microrod-like morphology into assembled nanoparticles. The average size of aggregated ZnO microstructures using psyllium husk is around 100–200 nm. The ZnO exhibits a wurtzite hexagonal crystalline phase even after the use of psyllium husk. The psyllium husk compresses two theta angles towards a low angle, which might create defects in the semiconducting material, whereby their role could be significant during the photodegradation process. Furthermore, the prepared ZnO microstructures were used for the methylene blue dye degradation under the illumination of UV light. The catalyst dose was also optimized and higher amount of photocatalyst show favorable photodegradation kinetics. The hybrid system reveals a photodegradation efficiency of 99.35%. The reaction kinetics were followed by the pseudo first order, and a faster rate towards degradation is noticed. Based on the obtained information, we believe that psyllium husk is a low-cost material, and offers two advantageous properties such as being a surface-modifying agent and allowing the simultaneous addition of carbon into ZnO, thereby facilitating the outperforming functionality of the ZnO-based photocatalyst for wastewater treatment.

## Figures and Tables

**Table 1:** The relative shift in two theta for psyllium husk assisted ZnO.

Sample	2θ		Peak Shift	FWHM	Average Grain Size (nm)
Pure ZnO	100	31.41679	0.31853	0.25691	25.3
	002	34.00772	0.23168	0.33262	
	101	35.88636	0.21821	0.44011	
Psyllium husk	100	31.09826		0.33231	24.1
	002	33.77604		0.29048	
	101	35.66815		0.4407	

**Table 2:** The calculated rate constants for the reaction kinetics of methylene blue degradation.

Catalyst Dose	Dye Conc.	Constant (K)	Dye Conc.	Constant (K)
5 mg		7.16 × 10^−3^ min^−1^		2.11 × 10^−2^ min^−1^
10 mg		7.52 × 10^−3^ min^−1^		2.19 × 10^−2^ min^−1^
15 mg	6.5 × 10^−5^ M	8.38 × 10^−3^ min^−1^	2.5 × 10^−5^ M	2.32 × 10^−2^ min^−1^
20 mg		9.59 × 10^−3^ min^−1^		2.62 × 10^−2^ min^−1^
Dye without catalyst	6.5 × 10^−5^ M	1.29 × 10^−3^ min^−1^		
Pristine ZnO	6.5 × 10^−5^ M	1.44 × 10^−3^ min^−1^		

**Table 3:** The comparative analysis of present work with published work.

Catalysts	Weight	Light Source	Dye Concentration	Time (min)	Dye Removal (%)	Method	Ref.
Macro/mesoporous anatase TiO_2_ ceramic	Eighty pieces (20 mg each piece)	UV light	RhB 10 mg mL^−1^	180	99.4%	A camphene-based freeze-casting process	[41]
TiO_2_ /Ficus carica leave extract	2 mg	Solar light	5 × 10^−5^ M (MB)	330	98.82%	Simple hydrothermal process	[42]
Fe_2_TiO_5_	50 mg	Sunlight	MB 10 mg L^−1^	250	97%	Sol–gel	[43]
ZnO @ S and Cl ion	15 mg	UV irradiation	1.87 × 10^−6^ M	150	100%	Simple hydrothermal process	[44]
Porous TiO_2_ ceramics	Eighty pieces (20 mg each piece)	UV light	RhB 10 mg L^−1^	300 min	99.3%	Camphene-based freeze-casting process	[45]
TiOSO_4_	(0.1 g)	UV irradiation	20 ppm MO	150	94%	Sol–gel method	[46]
ZnO @ W	20 mg	UV irradiation	250 mL MB	90	96.9%	Low-temperature aqueous chemical growth method	[47]
ZnO @Psyllium husk	20 mg	UV irradiation	2.5 × 10^−5^ M	150	99.35%	Simple hydrothermal process	Present work

## Data Availability

Not Applicable.
